# The Relationship Between Artificial Sweetener Intake from Soft Drinks and Internet Addiction Among Students: An Analytical and Cross-Sectional Study

**DOI:** 10.3390/ijerph22101554

**Published:** 2025-10-13

**Authors:** Nika Lovrincevic Pavlovic, Ivan Miskulin, Ivana Kotromanovic Simic, Marija Drmic, Marina Markovic, Ivana Milovanovic, Stela Jokic, Lana Radaus, Barbara Simatic, Maja Miskulin

**Affiliations:** 1 Faculty of Medicine Osijek, Josip Juraj Strossmayer University of Osijek, 31000 Osijek, Croatia; nika.felicita@gmail.com (N.L.P.); ivsimic@mefos.hr (I.K.S.); marija.drmic1@gmail.com (M.D.); marina.markovic0280@gmail.com (M.M.); ivana_-15@hotmail.com (I.M.); radaus.lana@gmail.com (L.R.); pejcic.barbara@gmail.com (B.S.); maja.miskulin@mefos.hr (M.M.); 2 Faculty of Food Technology Osijek, Josip Juraj Strossmayer University of Osijek, 31000 Osijek, Croatia; stela.jokic@ptfos.hr

**Keywords:** artificial sweeteners, artificially sweetened beverages, students, internet addiction, health, Croatia

## Abstract

The increasing consumption of artificially sweetened beverages among young people, coupled with prevalent digital technology use, presents growing public health concerns regarding potential effects on health and behavior. This study aimed to determine the concentrations of three commonly used artificial sweeteners—acesulfame K, saccharin, and aspartame—in soft drinks available on the market in Osijek, Croatia, to assess their compliance with European Union regulations, and to investigate the consumption patterns and possible associations with internet addiction among university students. Laboratory analysis of 43 beverages was performed using high-performance liquid chromatography with diode array detection, while a cross-sectional survey of 792 students collected data on sociodemographic characteristics, beverage consumption, and internet use. Acesulfame K was the most frequently detected sweetener, followed by aspartame and saccharin, with mean concentrations of 50.1 mg/L, 22.7 mg/L, and 19.76 mg/L, respectively. Overall, 85.7% of the students stated that they consumed artificially sweetened drinks, with an average consumption of 0.2 L/day. Internet addiction was found in 39.8% of the participants, but no significant correlation was found between beverage consumption and internet addiction (*p* = 0.177). All measured concentrations of sweeteners were below the legal limits. These results suggest that while exposure to artificial sweeteners in beverages is within safe limits, further research is needed to assess cumulative intake and its potential impact on behavioral health in young adults.

## 1. Introduction

The increasing consumption of artificially sweetened beverages (ASBs) and the pervasive use of digital technologies present growing challenges for public health, especially among younger populations [1]. These parallel behaviors have been independently associated with negative health effects and may interact in ways that lead to cumulative health burdens [2,3,4].

ASBs typically contain non-nutritional or artificial sweeteners (ASs) such as acesulfame K, saccharin, and aspartame that are marked as healthier sugar substitutes to reduce calorie intake [5]. However, recent studies have raised concerns about potential health risks of ASs, including type 2 diabetes, disruption of gut microbiota, cardiovascular complications, and adverse reproductive outcomes [6,7,8]. Their cumulative intake is of particular concern, especially given that many soft drinks (SDs) contain multiple ASs [9,10,11]. Findings from the NutriNet-Santé cohort indicate that over 50% of AS intake originates from ASBs, underscoring their significance as a primary exposure source [12]. Reports indicate that university students across diverse world regions exhibit consistently elevated SD consumption rates, reflecting a pervasive worldwide trend [13]. Recent studies also suggest that excessive consumption of ASBs among young individuals may have neurobehavioral consequences. For instance, aspartame has been linked to irritability and mood disturbances [14,15] while chronic intake of acesulfame K has been associated with impaired memory and altered neurometabolic functions in mice, indicating potential cognitive effects [16]. Additionally, long-term saccharin intake may lead to behavioral disruptions, oxidative stress, and brain tissue damage in rats [17].

Globally, high sugar-sweetened beverage (SSB) consumption among young adults increased from 6.6% in 1990 to 11.1% in 2021 [18]. According to one Norwegian study, ASB intake increased from childhood to adulthood as SSB consumption declined [19]. Among the most frequent reasons for increased consumption of ASBs among university students are health and weight management [20].

Furthermore, empirical evidence from university populations indicates that internet addiction (IA) is associated with unhealthy dietary behaviors, such as frequent snacking during online activity and increased consumption of carbonated beverages [21,22]. Several studies also suggest that excessive intake of SSBs and ASBs often co-occurs with prolonged screen time and problematic internet use [3,23,24,25], potentially reflecting shared behavioral patterns including a sedentary lifestyle, emotional eating, and exposure to food advertising [1].

Although direct studies linking IA and ABSs are scarce, evidence shows that excessive screen time—a common indicator of IA—is significantly associated with higher consumption of both SSBs and ASBs. It can therefore be argued that IA, as an extreme form of screen exposure, may represent a risk factor for increased intake of ASBs [26]. Taken together, these findings highlight the interconnection between internet-related behaviors and beverage consumption among students and highlight the importance of analyzing their beverage and lifestyle behaviors.

This study aimed to identify ASBs on the Osijek market containing acesulfame K, saccharin, or aspartame; quantify their concentrations and regulatory compliance; assess consumption among university students; estimate average AS intake; and examine potential associations between ASBs use and IA.

## 2. Materials and Methods

### 2.1. Study Design

This research consisted of two complementary phases: A laboratory analysis of ASBs available on the local market in Osijek and a cross-sectional survey assessing ASBs consumption patterns and potential IA among University students in Osijek. By integrating analytical chemistry and public health approaches, the study aimed to provide a comprehensive overview of AS content in ASBs and its potential behavioral correlates in a young adult population.

### 2.2. Laboratory Analysis of Artificial Sweeteners in Beverages

In March 2022, a total of 43 different ASBs—including fruit juices, fruit-based juices, beverages derived from plant extracts, and artificially flavored drinks such as energy drinks—were purchased from retail outlets in Osijek. Out of the 43 ASBs purchased, 38 contained acesulfame K, 19 contained saccharin, and 28 contained aspartame, indicating that several beverages included more than one type of sweetener.

The concentrations of acesulfame K, saccharin, and aspartame were determined using High-Performance Liquid Chromatography with a Diode Array Detector (HPLC-DAD) (Agilent Technologies Inc., Santa Clara, CA, USA) at the Laboratory of the Faculty of Food Technology, Osijek. Prior to analysis, samples were degassed using an Elma Elmasonic P 120 H ultrasonic bath (Elma Schmidbauer GmbH, Singen, Germany) and filtered through 0.2 µm nylon membranes. Both qualitative and quantitative assessments were carried out using an Agilent Technologies 1260 Infinity II HPLC system equipped with a quaternary pump, autosampler, thermostated column, and DAD (Agilent Technologies Inc., Santa Clara, CA, USA). ASs were identified and quantified through calibration with HPLC-grade standards obtained from Dr. Ehrenstorfer GmbH (Dr. Ehrenstorfer GmbH, Augsburg, Germany). All solvents used for the analysis were of analytical grade and purchased from J.T. Baker (J.T. Baker, Phillipsburg, NJ, USA). Chromatographic separation was achieved using a Zorbax Eclipse Plus C18 column, 10 × 4.6 mm, 5 µm particle size (Agilent Technologies Inc., Santa Clara, CA, USA), thermostated at 27 °C. The mobile phase was based on isocratic elution with phosphate buffer and acetonitrile at a flow rate of 1.5 mL/min. The system pressure was maintained at 145 bar, and analytes were detected at a wavelength of 210 nm. The injection volume was 20 µL, with a total runtime of 10 min per sample. Each sample was analyzed in triplicate to ensure accuracy and reproducibility.

### 2.3. Cross-Sectional Survey on ASBs Use and Internet Addiction

The second phase of the study was a cross-sectional survey aimed at examining patterns of ASB consumption and potential IA within the university population of Osijek. A total of 792 students aged 18 to 43 years, from various years and study programs across all higher education institutions in Osijek, participated in the survey. This sample size was considered representative of the broader university student population. Participation was voluntary and anonymous, with inclusion based on prior informed consent. Respondents who failed to provide consent or submitted incomplete questionnaires were excluded. Data were collected using a self-administered online questionnaire (Supplementary Material File S1), which took approximately 10 min to complete. It included 37 items covering sociodemographic data, ASB consumption, food allergies, and IA, which was assessed using the Young Internet Addiction Test (IAT), a widely used and validated measure of addictive internet use [27]. The IAT classifies users into four categories: normal internet use (0–30), mild addiction (31–49), moderate addiction (50–79), and severe addiction (80–100 points) [28].

The primary outcomes were ASB consumption and IA levels, assessed using the IAT. No clinically important difference was pre-defined, and no prognostic factors were controlled.

### 2.4. Statistical Analysis

Categorical variables were reported as absolute and relative frequencies. The Kolmogorov–Smirnov test was used to evaluate the normality of continuous variables. For normally distributed variables, data are expressed as mean ± standard deviation; for non-normally distributed variables, results are presented as median and interquartile range (IQR). Differences in categorical variables were tested using the chi-square test. All tests were two-tailed, and statistical significance was set at *p* < 0.05. Statistical analyses were performed using Statistica for Windows (version 10.0, StatSoft Inc., Tulsa, OK, USA).

## 3. Results

### 3.1. Concentrations of Artificial Sweeteners in Soft Drinks

A total of 24 commercially available brands of ASBs were included in the analysis, with multiple product variants represented across brands. The median values for acesulfame K, saccharin, and aspartame in all samples of ASBs that contained those substances were 50.1 mg/L, 19.76 mg/L, and 22.70 mg/L, respectively. The concentrations of these ASs in the analyzed beverages showed considerable variability, with acesulfame K present in the highest concentrations overall. Outliers were most prominent in the case of aspartame.

Among the 43 analyzed beverages, acesulfame K was detected in 38, aspartame in 28, and saccharin in 19 samples. Combinations of the three analyzed ASs were observed in the majority of samples. Specifically, 30 out of 43 beverages contained two or more of these sweeteners, while only 13 contained a single one.

The maximum permitted concentration (MPC) of acesulfame K in all types of SDs is 350 mg/L, while for aspartame it is 600 mg/L. In the case of saccharin, the MPC varies depending on beverage type: 80 mg/L in fruit juices and non-carbonated flavored beverages, and 100 mg/L in carbonated drinks. The measured concentrations of these three AS in ASB complied with MPC values as specified in Regulation (EC) No. 1333/2008 of the European Parliament and the Council on food additives within the European Union, except for artificial SDs, which are not regulated [29]. The measured concentrations of AS in the analyzed ASBs samples are represented in Table 1.

### 3.2. Soft Drink Consumption Among University Students

Out of 792 participants, 319 (40.3%) were male, while 473 (59.7%) were female. The median age of all respondents was 21.0 years, with an interquartile range from 20.0 to 22.0 years. A total of 37 responses were incomplete and therefore excluded from the statistical analysis. Regarding the distribution by study year, 170 participants (21.5%) were first-year students, while 226 (28.5%) were in their second year, which represented the largest group. Third-year students accounted for 188 (23.7%), followed by fourth-year students with 126 (15.9%) and fifth-year students with 79 (10%). Only 3 participants (0.4%) were sixth-year students, making it the smallest subgroup in the sample. The entire sample was drawn from a single institution, the Josip Juraj Strossmayer University of Osijek, with participants coming from 17 different Faculties and Departments to ensure broad representation of study fields across the university community. The majority of the participants were students from the Faculty of Medicine in Osijek, whereas the fewest participants were from the Department of Physics. Based on the field of study, participants were categorized into two groups: STEM disciplines (Science, Technology, Engineering, and Mathematics), with 513 participants (64.8%), and non-STEM disciplines, including 279 participants (35.2%). The highest number of participants were from biomedical studies (19.1%), while the lowest number came from arts and interdisciplinary fields (7.7%). In terms of academic progress, a total of 135 participants (17%) had repeated at least one academic year, whereas 657 (83%) had progressed through their studies without repeating a year. When asked about their prior education on nutrition and health, 505 participants (63.8%) stated that they had never received formal education on these topics, while 287 (36.2%) had some background knowledge in this area.

Participants also reported various living arrangements. Approximately 397 (50.1%) lived independently, while 230 (29%) resided with their parents or guardians. Additionally, 154 participants (19.4%) lived in a student dormitory, whereas only 11 (1.4%) lived with relatives. When examining employment status during studies, the majority of participants (556, or 70.2%) reported that they were not employed, whereas 236 (29.8%) were engaged in student jobs while pursuing their studies. Regarding student status, the vast majority (697 participants, 88%) were full-time students, while the remaining 95 (12%) were part-time students.

Regarding meal consumption habits, 437 participants (55.2%) reported eating meals at home, while 348 (43.9%) regularly ate in the student cafeteria. Only a small number, 7 participants (0.9%), stated that they frequently dined in restaurants.

When participants were asked about their consumption of SDs, 679 (85.7%) reported consuming various types of ASBs, whereas 113 (14.3%) indicated that they did not consume such beverages. Table 2 presents the distribution of respondents according to the type of ASBs they consume.

As for the circumstances of SD consumption, 332 participants (48.9%) reported consuming them after meals, while 222 (32.7%) consumed these beverages during social gatherings. Additionally, 80 participants (11.8%) consumed SDs while working on a computer, and 45 (6.6%) consumed them while watching television. Table 3 represents the distribution of participants based on the quantity of SDs consumed per day. Among consumers of SDs, the average daily intake was 0.2 L, corresponding to approximately one glass per day.

Adverse symptoms following the consumption of ASBs were reported by 99 participants (14.6%), while the 580 participants (85.4%) did not experience any such symptoms. Among all respondents, 730 participants (92.2%) stated that they do not have any food allergies, whereas 62 participants (7.8%) reported having a food allergy.

### 3.3. Internet Use and Its Association with ASBs Consumption

Regarding the primary reason for internet use, the majority of participants, 570 (72.0%), stated social media and entertainment as their main purpose. Additionally, 184 participants (23.2%) reported using the internet for academic or work-related needs, while 38 participants (4.8%) declared that they used the internet exclusively for playing online games. Exceeding normal internet use can lead to varying degrees of addiction, as reflected in the responses of our participants shown in Table 4.

According to the obtained results, 477 participants (60.2%) were not classified as internet-addicted (0–30 points), while the 315 participants (39.8%) exhibited some degree of IA (31 points or more).

Regarding the distribution by gender, out of 319 male students, 288 (90.3%) consumed some type of ASBs. In contrast, among 473 female students, 391 (82.7%) reported ASB consumption. The comparison of ASB consumption between genders revealed that male students consume ASBs significantly more frequently than female students (χ^2^ test; *p* = 0.003).

Concerning the association between IA and ASB consumption, the results indicate that 402 participants (59.2%) consumed ASBs while not being classified as internet-addicted (0–30 points). On the other hand, 277 participants (40.8%) were classified as having IA (31 points or more) while also consuming ASBs. Based on these findings, there was no statistically significant association between ASB consumption and IA among the student population in Osijek (χ^2^ test; *p* = 0.177).

When analyzing specific categories of SDs, the intake of ASs varies depending on the minimum (0 mg), average, and maximum ASB consumption levels, as detailed in Table 5.

## 4. Discussion

This study confirmed that concentrations of acesulfame K, saccharin, and aspartame in ASBs sold in Osijek are within EU regulatory limits. The same ASBs were consumed by 85.7% of students, with higher intake among males, while 39.8% exhibited some degree of IA. However, no significant association was found between ASB consumption and IA, and thus our initial hypothesis of a potential link was not supported.

### 4.1. Artificial Sweetener Concentrations and Regulatory Compliance

Among the analyzed beverages, acesulfame K was the most frequently detected sweetener, present in 88.4% of products, followed by aspartame (65.1%) and saccharin (44.2%). Fruit juices and fruit-based beverages were the most common product categories containing these sweeteners. The prevalence of ASs in these common product categories highlights a significant public health issue regarding consumer awareness, as many individuals, including young adults, do not associate fruit-based drinks with high levels of ASs. The measured median concentrations for all three sweeteners were consistent with those reported in prior European studies [30,31,32]. However, some regional variations may reflect differences in manufacturing practices and market trends. Due to the absence of quantitative labeling, a direct comparison between declared and measured sweetener concentrations was not possible. Nevertheless, all samples remained within the MPC values established under Regulation (EC) No. 1333/2008. Current EFSA acceptable daily intake (ADI) thresholds are set at 15 mg/kg bw/day for acesulfame K, 9 mg/kg bw/day for saccharin, and 40 mg/kg bw/day for aspartame [5]. The estimated daily intake based on average consumption (0.2 L) suggests that students’ exposure levels remain well below established ADI values. However, many beverages contained combinations of two or more ASs. The metabolic or behavioral effects of such synergistic exposure remain insufficiently understood and need further investigation. The complexity of those mixtures was confirmed by our laboratory analysis, which showed that 30 of the 43 beverages contained a combination of multiple ASs. Although this is a strong finding that could support the hypothesis of cumulative effects, a limitation of our cross-sectional design is that we could not differentiate the behavioral association for beverages with single vs. multiple ASs. However, given the high prevalence of these multi-AS products, it is highly plausible that the cumulative exposure to several ASs could have a more significant and detrimental influence on behavioral outcomes, including the risk and severity of IA. Furthermore, cumulative intake from multiple dietary sources such as processed foods, other beverages, and pharmaceuticals could also represent a potential public health concern [11].

### 4.2. Student Consumption Patterns and Estimated Daily Intake

Although average intake was 0.2 L per day, one-third of participants reported consuming two or more glasses daily. Based on these consumption patterns, average daily intakes were estimated at 10 mg for acesulfame K, 4.0 mg for saccharin, and 4.54 mg for aspartame—values that fall well within the established ADI thresholds, though continued moderate consumption is still recommended. Also, given that product labels do not provide quantitative information on sweetener content, consumer awareness and the ability to manage intake remain limited. Improved labeling regulations could enhance public health efforts. In addition, public perceptions of ASs are often inconsistent with regulatory conclusions. Despite widespread approval by food safety authorities, skepticism regarding the safety and long-term effects of ASs persists, particularly among health-conscious young adults. This perception may influence consumption choices and warrants further exploration in public health messaging [33].

The high prevalence of ASB consumption among students in Osijek reflects trends reported in both national and international studies [4,33,34]. As previously reported, young males generally consume larger amounts of SDs than their female counterparts [4,35]. According to Eurostat data, a higher proportion of men reported consuming SDs at least once per day—a pattern consistently observed across all EU member states [36]. Students in this study most frequently consumed ASBs after meals or during social activities, whereas fewer reported intake during screen-based activities. These results reflect broader lifestyle patterns, particularly among digital-native populations, where beverage consumption often overlaps with extended digital media use [37].

### 4.3. Internet Addiction and Behavioral Associations

Although IA was observed in nearly 40% of respondents, no statistically significant association with ASB consumption was found. This finding does not align with earlier evidence linking SSBs to problematic internet use, particularly among adolescents [23,24]. The observed discrepancy may stem from behavioral or neurochemical differences between caloric and non-caloric sweeteners.

The absence of a statistically significant correlation between ASB consumption and IA in our study represents a notable contrast to recent international literature. Other researchers often find clear behavioral mechanisms linking compulsive internet use to disordered eating habits. A recent study conducted on a student population in Turkey found that with increasing levels of IA, the risk of eating disorders, anxiety due to obesity, and excessive interest in losing weight increases. Furthermore, problematic internet users were classified as obese, while the vast majority confirmed that visual content and the act of using the internet directly influence their food choices [38]. A related European study demonstrated a correlation between prolonged screen time and higher intake of sweetened carbonated beverages among their participants [37].

Consequently, the lack of a statistical association between ASB consumption and IA in our study can best be explained by the specifics of our sample, rather than by the absence of a general association. Despite evidence suggesting that IA may induce changes such as reduced meal size and loss of appetite [39], our sample may not have had a high enough average dose of ASB (0.2 L/day) or intensity of IA to reflect this behavioral effect in a significant correlation. Notably, existing literature has primarily focused on SSBs while ASBs remain understudied in this context. Still, given that ASBs are frequently consumed during screen-based activities, and that both behaviors stimulate brain regions associated with instant pleasure [40], it is plausible that shared mechanisms exist. These parallels highlight the need for integrative studies addressing co-occurring lifestyle behaviors in youth. In light of the increasing substitution of sugar with ASs in global beverage markets, continued surveillance of cumulative AS intake across demographic groups is warranted. Future research should also examine whether early exposure to ASBs influences sweetness perception and long-term consumption patterns. Finally, it must be acknowledged that the cross-sectional design of our study cannot determine whether IA causes ASB consumption or vice versa, which is a methodological limitation consistently highlighted in similar studies.

Interestingly, 14.6% of participants reported experiencing adverse symptoms following ASB consumption, though the nature of these symptoms was not specified. Because of this, it is difficult to draw any medical conclusions, but the findings may indicate that some individuals are more sensitive to ASs. Further clinical and sensory studies are needed to clarify these effects.

### 4.4. Limitations

This study has several limitations. The results are based on a student sample from Osijek and are not generalizable to other demographic groups or regions. Self-reported data introduce the possibility of recall bias regarding beverage intake and screen use. Due to the cross-sectional study design, causal relationships between ASB consumption and IA cannot be established. The scope of laboratory testing was limited to products available at the time of sampling and did not cover all possible sources of AS intake. Furthermore, our survey design lacked some details (e.g., specific brand or ingredient profiles) to correlate individual self-reported symptoms directly with the precise AS composition of the consumed beverages. This could be important information for targeted public health advice, due to the possible connection between adverse reactions and intake of a specific sweetener. Additionally, confounding factors such as dietary patterns, physical activity, and mental health were not controlled for and may have influenced both ASB consumption and internet use, potentially affecting the study results. Longitudinal and experimental studies are necessary to better understand the long-term health effects of AS consumption and its behavioral correlates.

## 5. Conclusions

While ASB consumption complied with regulatory limits and remained within ADI thresholds, its high prevalence alongside intensive internet use suggests potential behavioral implications. Although no significant association with IA was found, possible links in specific subgroups or contexts cannot be ruled out. Future research should address cumulative sweetener exposure and its interaction with digital behaviors through longitudinal studies. Furthermore, future studies should specifically investigate whether exposure to complex sweetener profiles (i.e., ‘Combo’ vs. ‘AS-Only’ and single vs. multiple ASs) is differentially associated with behavioral outcomes like IA. Additionally, detailed prospective consumption diaries should be employed to accurately link self-reported adverse symptoms to the specific analytical composition of consumed beverages, thus clarifying the issue of individual sensitivity.

## Figures and Tables

**Table 1:** Measured concentrations (mg/L) of ASs in analyzed ASBs.

Number	Brand	Product Type	Acesulfam K	Saharin	Aspartame
1.	1	a	83.27	19.76	-
2.		b	38.41	-	-
3.		c	-	52.41	-
4.	2	a	-	16.31	-
5.		b	167.24	-	-
6.		c	80.58	16.72	-
7.	3	a	62.7	-	40.20
8.	4	a	58.67	15.57	6.08
9.		b	2.26	19.32	5.04
10.		c	19.705	23.15	6.83
11.		d	9.804	18.51	7.91
12.		e	47.38	19.72	4.53
13.	5	a	19.69	23.15	2.37
14.	6	a	172.18	-	73.76
15.	7	a	149.94	-	123.04
16.		b	183.68	-	120.37
17.	8	a	41.99	36.41	14.98
18.	9	a	126.76	-	55.84
19.	10	a	14.32	-	-
20.		b	52.84	-	29.39
21.	11	a	-	13.16	-
22.		b	20.86	-	15.39
23.		c	23.00	-	14.592
24.	12	a	202.38	-	161.86
25.	13	a	152.25	-	97.49
26.	14	a	91.20	-	106.64
27.	15	a	26.28	51.20	19.34
28.	16	a	44.40	-	-
29.	17	a	28.73	36.71	6.025
30.		b	17.08	33.31	15.73
31.		c	15.13	30.53	14.91
32.	18	a	21.45	69.64	22.01
33.	19	a	31.685	-	226.21
34.		b	38.37	-	415.35
35.	20	a	165.42	-	78.49
36.		b	168.58	-	83.37
37.	21	a	183.57	-	-
38.		b	217.18	-	-
39.		c	57.16	-	-
40.	22	a	26.25	-	-
41.	23	a	55.40	-	60.61
42.	24	a	-	7.08	-
43.		b	-	2.32	-

**Table 2:** Types of artificially sweetened beverages consumed by students.

Type of Artificially Sweetened Beverage	Number of Participants (%)
Fruit juices	322 (47.4%)
Fruit-based beverages	218 (32.1%)
Beverages made from plant extracts	82 (12.1%)
Artificially flavored soft drinks	57 (8.4%)

**Table 3:** Distribution of daily intake volumes of artificially sweetened beverages among students.

Quantity of Soft Drinks Consumed	Number of Participants (%)
0 L (0 glasses)	78 (11.5%)
0.2 L (1 glass)	373 (54.9%)
0.5 L (2 glasses)	152 (22.4%)
0.75 L (3 glasses)	50 (7.4%)
1 L (4 glasses)	26 (3.8%)

**Table 4:** Classification of students by level of internet addiction according to the internet addiction test.

Internet Addiction Category	Number of Participants (%)
No addiction (0–30 points)	477 (60.2%)
Mild addiction (31–49 points)	217 (27.4%)
Moderate addiction (50–79 points)	94 (11.9%)
Severe addiction (80–100 points)	4 (0.5%)

**Table 5:** Estimated average and maximum daily intake of artificial sweeteners by beverage type.

Artificial Sweetener		Daily Consumption (mg)	Average (1 Glass~0.2 L)	Maximum (4 Glasses~1.0 L)
	Type and Number of Soft Drink			
Acesulfame K	Fruit juices (12)		21.9	87.7
	Fruit-Based Beverages (9)		152.3	609.0
	Plant Extract Juices (8)		33.6	134.3
	Artificial flavored beverages (9)		149.9	599.8
Saccharin	Fruit juices (8)		3.9	19.5
	Fruit-Based Beverages (3)		3.3	16.7
	Plant Extract Juices (6)		7.0	35.0
	Artificial flavored beverages (2)		0.9	4.7
Aspartame	Fruit juices (11)		1.58	6.33
	Fruit-Based Beverages (7)		83.37	333.48
	Plant Extract Juices (5)		15.73	62.92
	Artificial flavored beverages (5)		120.37	481.48

## Data Availability

The data supporting the findings of this study are not publicly available due to confidentiality, but are available from the corresponding author upon reasonable request and in line with ethical and privacy considerations.

## Supplementary Materials

The following supporting information can be downloaded at: https://www.mdpi.com/article/10.3390/ijerph22101554/s1, File S1: Questionnaire on the influence of soft drinks on students’ health.
