# Supplementary material for: The Relationship Between Artificial Sweetener Intake from Soft Drinks and Internet Addiction Among Students: An Analytical and Cross-Sectional Study
Source: Int J Environ Res Public Health. 2025 Oct 13;22(10):1554. doi: 10.3390/ijerph22101554 (PMC12563211; doi:10.3390/ijerph22101554)
Supplement: Supplementary file 1 [file ijerph-22-01554-s001.zip › ijerph-3885498-supplementary.pdf]

QUESTIONNAIRE ON THE INFLUENCE OF SOFT DRINKS ON STUDENTS' HEALTH

Please circle your answer or write it on the line!

1. Gender M F

2. Age _________ (years)

3. Year of study: 1 2 3 4 5 6

4. Faculty:

a) Faculty of Medicine Osijek
b) Faculty of Dental Medicine and Health
c) Faculty of Food Technology Osijek
d) Faculty of Agrobiotechnical Sciences Osijek
e) Department of Biology
f) Department of Chemistry
g) Department of Physics
h) Department of Mathematics
i) Faculty of Kinesiology
j) Faculty of Economics Osijek
k) Faculty of Education
l) Faculty of Law Osijek
m) Faculty of Humanities and Social Sciences
n) Catholic Faculty of Theology
o) Academy of Arts and Culture
p) Faculty of Electrical Engineering, Computer Science and Information Technology Osijek
q) Faculty of Civil Engineering and Architecture

5. Have you ever repeated a study year? YES NO

6. Have you learned about the impact of nutrition on health at your faculty? YES NO

7. During your studies you live:

a) with parents/guardians
b) independently in a rented apartment
c) with relatives
d) in a student dormitory

8. Do you work during your studies? YES NO

9. Your student status is:

a) full-time student
b) part-time student

10. Place where you most often consume meals:

a) Home
b) Restaurants
c) Student canteen

11. Do you consume soft drinks? YES NO

12. Which soft drink do you consume most often?
a) fruit juices (e.g. thick peach/apricot juice, apple/orange juice)
b) fruit-based beverages (e.g. Fanta, Coca-Cola, Sprite, Cockta, Pepsi Cola)
c) plant extract juices (e.g. various types of Schweppes, various types of iced tea)
d) artificial flavored beverages (e.g. Iso sport, Red Bull, Monster Energy, etc.)

13. The average daily amount of soft drinks you consume is:

a) 0 l (0 glasses)
b) 0.2 l (1 glass)
c) 0.5 l (2 glasses)
d) 0.75 l (3 glasses)
e) 1 l (4 glasses)

14. In which circumstances do you most often consume soft drinks:

a) while going out
b) after meals
c) while working on the computer
d) while watching TV

15. Are you allergic to any type of food, and if yes, to which? a) YES ___________ b) NO

16. After consuming soft drinks, have you ever had any of the following symptoms: drowsiness, headache, fatigue, dizziness, vomiting, rapid heartbeat, lightheadedness, irritability, restlessness, memory loss, blurred vision, rash, increased appetite? YES NO

17. I use the Internet mostly for:

a) faculty/work purposes
a) social networks (Facebook, Twitter, Instagram…) and entertainment
b) online games

Please circle the number 0-5 to answer the following questions, where the meaning of the numbers is as follows:

0-NOT APPLICABLE; 1- RARELY; 2-OCCASIONALLY; 3-FREQUENTLY; 4- OFTEN; 5-ALWAYS

Question		Scale					
1.	How often do you stay online longer than you intended?	0	1	2	3	4	5
2.	How often do you neglect household chores to spend more time online?	0	1	2	3	4	5
3.	How often do you prefer the excitement of the Internet to intimacy with your partner?	0	1	2	3	4	5
4.	How often do you form new relationships with fellow online users?	0	1	2	3	4	5
5.	How often do others complain about the time you spend online?	0	1	2	3	4	5
6.	How often do your grades or school work suffer because of the amount you spend online?	0	1	2	3	4	5
7.	How often do you check your email before something else that you need to do?	0	1	2	3	4	5
8.	How often does your job performance or productivity suffer because of the Internet?	0	1	2	3	4	5
9.	How often do you become defensive or secretive when anyone asks you what you do online?	0	1	2	3	4	5
10.	How often do you block out disturbing thoughts about your life with soothing thoughts of the Internet?	0	1	2	3	4	5
11.	How often do you find yourself anticipating when you will go online again?	0	1	2	3	4	5
12.	How often do you fear that life without the Internet would be boring, empty, and joyless?	0	1	2	3	4	5
13.	How often do you snap, yell, or act annoyed if someone bothers you while you are online?	0	1	2	3	4	5
14.	How often do you lose sleep due to being online?	0	1	2	3	4	5
15.	How often do you feel preoccupied with the Internet when off-line, or fantasize about being online?	0	1	2	3	4	5
16.	How often do you find yourself saying "just a few more minutes" when online?	0	1	2	3	4	5
17.	How often do you try to cut down the amount of time you spend online and fail?	0	1	2	3	4	5
18.	How often do you try to hide how long you've been online?	0	1	2	3	4	5
19.	How often do you choose to spend more time online over going out with others?	0	1	2	3	4	5
20.	How often do you feel depressed, moody, or nervous when you are off-line, which goes away once you are back online?	0	1	2	3	4	5

THANK YOU FOR YOUR PARTICIPATION!
